# Treatment initiation among persons diagnosed with drug resistant tuberculosis in Johannesburg, South Africa

**DOI:** 10.1371/journal.pone.0181238

**Published:** 2017-07-26

**Authors:** Denise Evans, Kathryn Schnippel, Caroline Govathson, Tembeka Sineke, Andrew Black, Lawrence Long, Rebecca Berhanu, Sydney Rosen

**Affiliations:** 1 Health Economics and Epidemiology Research Office, Department of Internal Medicine, School of Clinical Medicine, Faculty of Health Sciences, University of the Witwatersrand, Johannesburg, South Africa; 2 Right to Care, Johannesburg, South Africa; 3 Clinical HIV Research Unit, Department of Internal Medicine, School of Clinical Medicine, Faculty of Health Sciences, University of the Witwatersrand, Johannesburg, South Africa; 4 Wits Reproductive Health and HIV Institute, Department of Internal Medicine, School of Clinical Medicine, Faculty of Health Sciences, University of the Witwatersrand, Johannesburg, South Africa; 5 Division of Infectious Diseases, University of North Carolina, Chapel Hill, NC, United States of America; 6 Department of Global Health, Boston University School of Public Health, Boston, MA, United States of America; Fundació Institut d’Investigació en Ciències de la Salut Germans Trias i Pujol, Universitat Autònoma de Barcelona, SPAIN

## Abstract

**Background:**

In South Africa, roughly half of the drug-resistant TB cases diagnosed are reported to have been started on treatment. We determined the proportion of persons diagnosed with rifampicin resistant (RR-) TB who initiated treatment in Johannesburg after the introduction of decentralized RR-TB care in 2011.

**Methods:**

We retrospectively matched adult patients diagnosed with laboratory-confirmed RR-TB in Johannesburg from 07/2011-06/2012 with records of patients initiating RR-TB treatment at one of the city’s four public sector treatment sites (one centralized, three decentralized). Patients were followed from date of diagnosis until the earliest of RR-TB treatment initiation, death, or 6 months’ follow-up. We report diagnostic methods and outcomes, proportions initiating treatment, and median time from diagnosis to treatment initiation.

**Results:**

594 patients were enrolled (median age 34 (IQR 29–42), 287 (48.3%) female). Diagnosis was by GenoType MTBDRplus (Hain-Life-Science) line probe assay (LPA) (281, 47.3%), Xpert MTB/RIF (Cepheid) (258, 43.4%), or phenotypic drug susceptibility testing (DST) (30, 5.1%) with 25 (4.2%) missing a diagnosis method. 320 patients (53.8%) had multi-drug resistant TB, 158 (26.6%) rifampicin resistant TB by Xpert MTB/RIF, 102 (17.2%) rifampicin mono-resistance, and 14 (2.4%) extensively drug-resistant TB. 256/594 (43.0%) patients initiated treatment, representing 70.7% of those who were referred for treatment (362/594). 338/594 patients (57.0%) did not initiate treatment, including 104 (17.5%) who died before treatment was started. The median time from sputum collection to treatment initiation was 33 days (IQR 12–52).

**Conclusion:**

Despite decentralized RR-TB treatment, fewer than half the patients diagnosed in Johannesburg initiated appropriate treatment. Offering treatment at decentralized sites alone is not sufficient; improvements in linking patients diagnosed with RR-TB to effective treatment is essential.

## Introduction

In 2015, a global total of 132 120 cases of multi-drug resistant tuberculosis (MDR-TB) and rifampicin resistant TB (RR-TB) were notified to the World Health Organization (WHO). This represented 23% of the estimated 580 000 cases of MDR/RR-TB cases worldwide demonstrating a major diagnostic gap [1].

South Africa, with less than 1% of the world’s population, accounted for 15% of the notified cases of MDR/RR-TB globally, with 19 613 cases of laboratory confirmed MDR/RR-TB cases in 2015 [1]. Although the proportion of eligible patients who initiated MDR-TB treatment in South Africa increased from 41% in 2013 to 64% in 2015, a major diagnosis-to-treatment gap remains [1,2]. Despite this increase, the proportion initiating MDR-TB treatment in South Africa is below the global figure of 90% [3]. There is no possibility of achieving the Global Plan to End TB by 2020 if a third of patients diagnosed with MDR/RR-TB never start treatment [1].

Prior to 2011, all patients with RR-TB in South Africa (a category that includes rifampicin resistant TB with unknown additional drug resistance, MDR-TB, and extensively drug resistant TB (XDR) TB were treated at specialized, inpatient facilities for the duration of the intensive phase of drug resistant (DR-) TB treatment, typically six months [4]. Studies reported that time from sputum collection to inpatient admission ranged from 10–16 weeks, and up to 40% of MDR-TB patients died within 30 days of sputum collection in certain provinces [5–7]. In 2011, South Africa improved its ability to test for DR-TB by introducing Xpert MTB/RIF (Cepheid), a molecular test capable of identifying both TB and rifampicin resistance in under two hours [8,9]. At the same time, in order to increase treatment capacity, minimize treatment delays, and improve outcomes, the South African National TB program announced a framework for “decentralized and deinstitutionalized management” of MDR-TB, authorizing outpatient initiation of DR-TB treatment [10]. This policy allows patients to start treatment at sites closer to their homes and remain resident at home for the duration of treatment, rather than being isolated at one of the country’s few specialized, provincial-level inpatient TB hospitals. Following implementation of the new policy, the number of sites initiating DR-TB treatment quadrupled nationally, with at least one treatment site in each district [11].

To help inform further improvements in DR-TB programs and guidelines, we evaluated the extent to which better diagnosis with Xpert MTB/RIF and decentralized service delivery has improved DR-TB treatment initiation in South Africa. We conducted a retrospective medical register review to match patients diagnosed with laboratory-confirmed RR-TB, as reported to the City of Johannesburg in Gauteng Province, to DR-TB treatment initiation records at the city’s four public sector treatment sites during the study period. We report diagnostic methods and outcomes, proportions initiating treatment, and median time from diagnosis to treatment initiation.

## Methods

### Setting, sites, and population

We conducted a retrospective medical register review of adult (≥18 years) patients with laboratory-confirmed RR-TB between July 2011 and June 2012 in the City of Johannesburg (COJ), the largest metropolitan area in the country. By June/July 2012 the National Health Laboratory Service Laboratory (NHLS) had performed 54,232 Xpert MTB/RIF tests in Gauteng Province [12]. Of these MTB was detected in 12.6% (n = 6,857) and 6.67% of these were resistant to rifampicin (n = 457). Based on the NHLS data the City of Johannesburg, which has an estimated overall HIV prevalence of 11.1%, reported 42,924 Xpert MTB/RIF tests between July 2011 and June 2012 [13].

**Fig 1:**
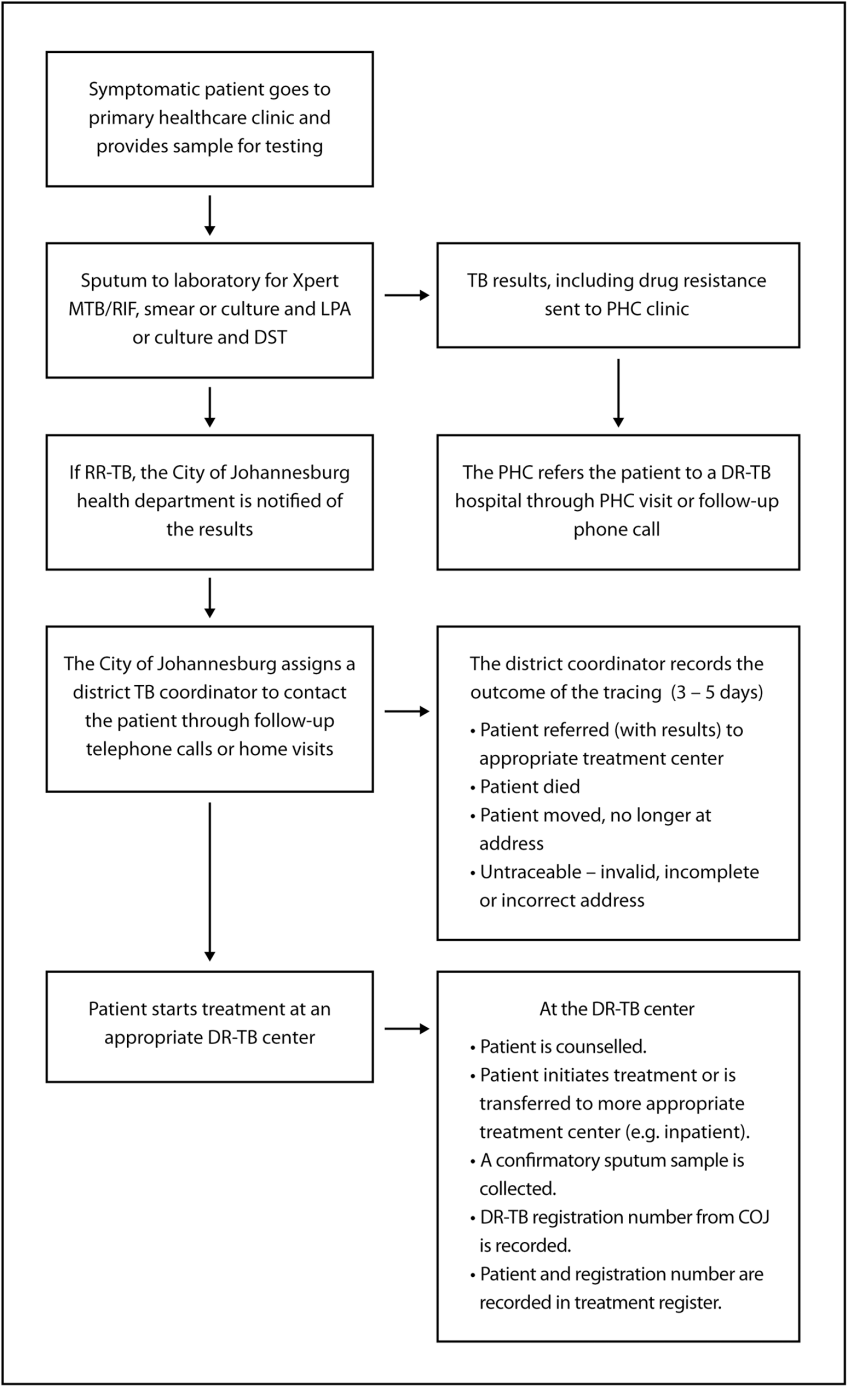
Schematic of the procedures and structures established within district health system for the follow-up of all patients diagnosed with RR-TB.

As illustrated in Fig 1, symptomatic patients presenting at one of the city’s primary healthcare clinics provide 1–2 sputum samples, which are sent to the NHLS for the diagnosis of TB and rifampicin resistance or multi-drug resistance (rifampicin and isoniazid resistance). The NHLS sends all RR-TB results back to the diagnosing clinic and to the COJ TB coordinator, where the results are recorded and a DR-TB case registration number is assigned. The COJ assigns each patient to a district TB coordinator who contacts and refers the patient to appropriate care and reports the outcome of the tracing to the COJ within 3–5 days. At the diagnosing clinic, a nurse records the results in the TB suspect register and refers the patient to an appropriate DR-TB treatment center. Once the patient arrives at the DR-TB treatment center, the patient is initiated onto DR-TB treatment and the DR-TB treatment center records the patient information and DR-TB case registration number (obtained from COJ). Alternatively, the DR-TB treatment center may decide to transfer the patient to a more appropriate facility (e.g. if the first DR-TB treatment center is an outpatient clinic and the patient should be admitted as an inpatient). The COJ maintains an electronic register of diagnosed patients and also has paper records of tracing activities.

During the study period, DR-TB patients within COJ could be referred to one inpatient treatment center or one of three outpatient treatment centers. Sizwe Tropical Disease Hospital (STDH), the inpatient treatment center, serves as a referral center for all DR-TB in Gauteng Province, where Johannesburg is located [14]. Prior to decentralization in 2011, all MDR-TB and XDR-TB patients in the province were treated at STDH; currently all XDR-TB patients are still treated there. Between January 2007 and December 2010 a total of 2005 MDR-TB patients were admitted to STDH [14]. Records for patients initiated onto treatment for MDR-TB and XDR-TB at STDH are entered directly into the national electronic DR-TB register, which is called EDRweb.

In 2011, three outpatient clinics, at Charlotte Maxeke Johannesburg Academic Hospital (CMJAH), Helen Joseph Hospital (HJH), and South Rand Hospital (SRH), were authorized to initiate and treat DR-TB on an outpatient basis. Eligibility criteria for outpatient initiation of DR-TB treatment were established by the National Department of Health and take into account transmission risk (smear negative or extra-pulmonary TB), clinical condition (e.g. body mass index >18.5 kg/m^2^), social support, and stable accommodation [10]. Patients are also treated at the outpatient facility if there are no beds available at STDH or if the patient refuses to be admitted for inpatient care. All three facilities maintain on-site electronic clinical patient DR-TB registers.

We enrolled in the study all adults (18 years and older) who had a diagnosis of laboratory-confirmed RR-TB reported to the COJ between July 2011 and June 2012. We excluded patients who enrolled in care at one of the four DR-TB treatment sites after transfer from another district, province, or country.

### Diagnostic algorithms

Xpert MTB/RIF was implemented as the first-line diagnostic test for tuberculosis in South Africa during the study period. Prior to 2011, the TB diagnostic algorithm relied on smear microscopy for cases of suspected tuberculosis. Line probe assay (LPA; GenoType MTBDRplus Hain-Life-Science) with culture and phenotypic DST were only done in cases of suspected drug-resistance such as contact of person with known DR-TB; relapse and treatment failure [15]. With implementation of Xpert MTB/RIF universal DST for rifampicin became the standard of care. Xpert MTB/RIF roll-out by the National Health Laboratory Services (NHLS) in the City of Johannesburg began in August 2011 with full utilization by January 2012 [12]. Certain sites had earlier access to Xpert MTB/RIF prior to implementation by the NHLS through validation research studies.

### Outcomes, data, and data analysis

The primary outcome of the study was the proportion of patients who initiated DR-TB treatment at one of the city’s four treatment sites within six months of sputum collection. To determine who initiated DR-TB treatment we matched eligible patients in the COJ register to electronic registers at the sites. Study staff worked with facilities and the COJ to verify and correct patient information (e.g. to correctly record DR-TB registration numbers in facility registers and query specific cases where diagnosis date or treatment initiation date preceded the sputum collection date). For patients in the COJ register who could not be matched to the electronic registers by DR-TB case registration number or where the registration number was missing, probabilistic matching using first name, surname, date of birth, and sex was used to match individuals. For patients who still could not be matched, we then matched on (i) first three initials of name and surname, date of birth and sex, (ii) first three initials of name and surname, year of birth and sex, and (iii) a four-character code created using a phonetic algorithm (Soundex) to account for minor differences in spelling (e.g. ‘Ngcobo’ vs ‘Ngobo’). Matched pairs were manually checked and verified by two independent evaluators. DR-TB treatment initiation date was obtained from the electronic registers at the sites for patients who could be matched.

The outcomes of COJ tracing for all patients diagnosed with RR-TB were obtained from the COJ register and were defined as died, moved or transferred out of the city, lost (untraceable), or successfully traced and referred.

Variables including first name, surname, date of birth, sex, tracing outcome, treatment initiation date, and address were obtained from the COJ electronic register. In addition, variables collected for each patient such as disease classification, diagnosis method and diagnosis date (obtained from NHLS), smear microscopy result, and site of disease were also obtained from the registers. We further classified RR-TB with unknown or pending sensitivities to other drugs according to the diagnosis method used: Xpert MTB/RIF, LPA or phenotypic DST. In addition, we used the address the patient recorded in the COJ electronic register to calculate the distance from the diagnosing/referring clinic to the patient’s residence and, for those initiating treatment, from the patient’s residence to the DR-TB treatment center using Google Maps and SAS version 9.3. We further divided distance from the patient’s residence to diagnosing/referring clinic into quintiles. The first and second quintile were groups and labeled “near” (<11.2 km), the third labelled intermediate (11.2–54.3 km) and the third to fifth quintile were grouped and labelled far (≥ 54.3 km) [16–18].

In the analysis, demographic and clinical characteristics are presented using proportions for categorical variables and medians with corresponding interquartile ranges (IQR) for continuous variables. We first estimated the proportion of the population who experienced the primary outcome. We used competing risk regression method from Fine and Gray to fit a proportional subdistribution hazard model [19,20]. Death was considered a competing risk, which arises when the event of interest (initiation of DR-TB treatment) can be impeded by a prior event of a different type (e.g. death). Patient time accrued from the date of diagnosis until the earliest of DR-TB treatment initiation, death, lost/transferred out of the city/moved, or 6 months’ follow-up after diagnosis. In instances where the transfer out date was missing, the date of last contact with the patient, as recorded by the TB district coordinator, was used as the outcome date. Subdistribution hazard ratios (sHR) with 95% confidence intervals (CI) are presented. Categorized age, sex, disease classification and diagnosis method were included in the adjusted model along with other *a priori* identified characteristics.

To identify predictors of all-cause mortality, we used Cox proportional hazard regression to estimate a hazard ratio (HR) and corresponding 95% confidence interval (CI). Patient time accrued from the date of diagnosis until the earliest of all-cause mortality, lost (untraceable), transferred out of the city/moved, or 6 months’ follow-up after diagnosis. Crude and adjusted hazard ratios (HR) with 95% confidence intervals (CI) are presented.

We also compared median time from sputum collection to treatment initiation by disease classification, site of treatment initiation (decentralized-outpatient vs centralized-inpatient) and diagnosis method using the student t test for parametric or Kruskal-Wallis for non-parametric data. We also tested the association between treatment site and initiating treatment within five days of diagnosis using log-binomial regression with crude risk ratios and 95% confidence intervals. All analyses were carried out using SAS version 9.3 (SAS Institute, Cary, North Carolina, USA).

This study was approved by the Human Research Ethics Committee (Medical) of the University of the Witwatersrand (Wits HREC M130601). Participants did not provide written or verbal consent to participate in the study as all data analyzed were collected as part of routine diagnosis and treatment.

## Results

We enrolled 594 patients in the study. As described in Table 1, they had a median (IQR) age of 34 (29–42) years, and 48.3% were female. Most were diagnosed by LPA (281, 47.3%) or Xpert MTB/RIF (258, 43.4%), with a few by phenotypic DST (30, 5.1%) or by unknown (missing) diagnostic method (25, 4.2%). 320 patients (53.8%) had MDR-TB, 158 (26.6%) had rifampicin resistant TB by Xpert MTB/RIF with no additional drug susceptibility results available, 102 (17.2%) had rifampicin mono-resistance, and 14 (2.4%) had XDR TB.

**Table 1:** Demographic and clinical characteristics of patients who had a diagnosis of laboratory-confirmed RR-TB reported to the COJ for tracing between July 2011 and June 2012 (n = 594).

Characteristic	Description		N = 594
Gender	Male	n,%	307 (51.7%)
	Female		287 (48.3%)
Age, years		Median, IQR	34 (29–42)
	< 30	n, %	162 (27.3%)
	30–45		308 (51.9%)
	45–60		105 (17.7%)
	≥ 60		19 (3.2%)
Disease classification	RR-TB by Xpert^	n, %	158 (26.6%)
	RR-TB (mono and poly)^^		102 (17.2%)
	MDR-TB		320 (53.8%)
	XDR TB		14 (2.4%)
Diagnosis method	Xpert MTB/RIF	n, %	258 (43.4%)
	GenoType MTBDRplus line probe assay		281 (47.3%)
	Phenotypic drug susceptibility testing		30 (5.1%)
	Unknown		25 (4.2%)
AFB smear microscopy status	Positive	n, %	144 (24.2%)
	Negative		78 (13.1%)
	Unknown		372 (62.7%)
Site of disease	Pulmonary	n, %	581 (97.8%)
	Extra-pulmonary		13 (2.2%)
Treatment site	Helen Joseph Hospital (outpatient)	n, %	70/256 (27.3%)
	Charlotte Maxeke Hospital (outpatient)		61/256 (23.8%)
	South Rand Hospital (outpatient)		37/256 (14.5%)
	Sizwe Tropical Disease Hospital (inpatient)		88/256 (34.4%)
Location of residence	Distance from diagnosing/referral facility to residence, km (n = 402; 67.7%)	Median, IQR	13 (11.2–54.3)
	Distance from DR-TB treatment facility to residence, km (n = 249; 97.3%)	Median, IQR	11.2 (7.7–23.0)

RR-TB rifampicin resistant tuberculosis; MDR-TB multi-drug resistant TB; XDR TB extensively drug resistant TB; km kilometer; AFB acid fast bacilli; IQR inter-quartile range

^RR-TB diagnosed by Xpert with unknown or pending sensitivities to other drugs

^^mono- or poly-resistant is resistance to rifampicin alone or rifampicin plus another first-line drug (other than isoniazid), confirmed by LPA or DST.

**Fig 2:**
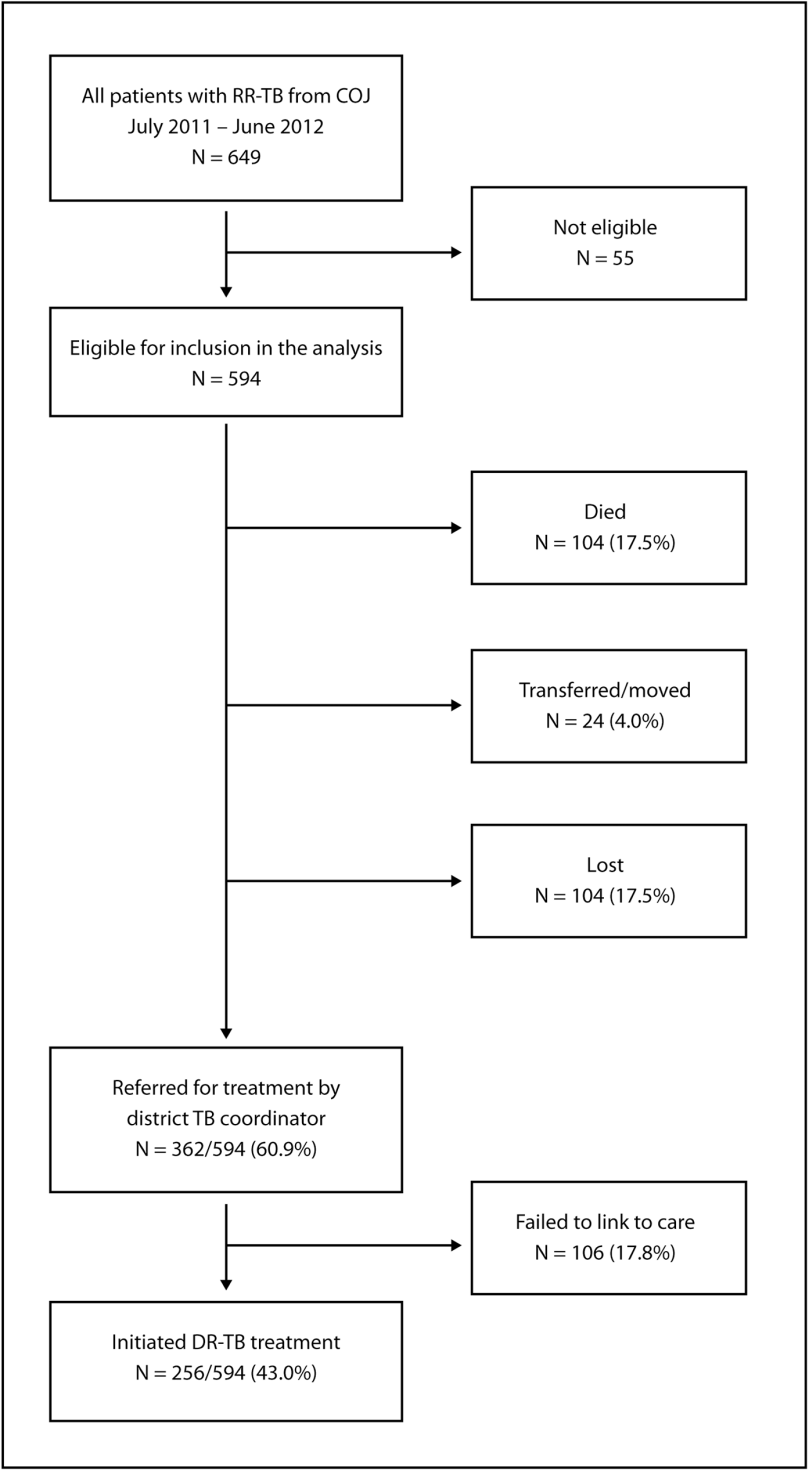
Schematic showing the proportion of those diagnosed with RR-TB in the COJ who were successfully traced and referred and who initiated treatment along with other tracing outcomes including death, moved or transferred out of the city and untraceable.

Only 43% (256/594) of the patients diagnosed with DR-TB in COJ initiated treatment at one of the four treatment sites within six months of diagnosis. Of the 594 laboratory confirmed cases reported to the COJ, 60.9% (362/594) were successfully traced and referred for treatment; the 256 who actually started treatment represent 70.7% of these (Fig 2). Among the 338 patients (57.0%) who did not initiate treatment, 104 died before treatment was started, 24 transferred out or moved out of the province and 104 could not be traced (lost). Of the 362 patients who were successfully traced and referred, 106 failed to link to care after referral. According to the COJ tracing outcome, median time from sputum collection to death was 19 days (IQR 10–30) and from sputum collection to other reported outcome (lost/untraceable or transferred out of the city/moved) was 14 days (IQR 8–34).

Among those who did start treatment, 34.4% (88/256) initiated treatment at the centralized-inpatient DR-TB treatment center, and 65.6% (168/256) initiated at one of the three decentralized-outpatient sites. Demographic and clinical characteristics of those who initiated treatment are presented in Table 2.

**Table 2:** Comparison of case characteristics between those initiated treatment at outpatient and inpatient sites (n = 256).

Characteristic	Description		Outpatient sites (n = 168)	Inpatient site (n = 88)	P value
Sex	Female	n,%	89 (53.0%)	41 (46.6%)	0.332
Age, years		Median, IQR	34 (29–42)	35 (29–44)	0.773
	< 30	n, %	49 (29.2%)	24 (27.3%)	
	30–45		83 (49.4%)	42 (47.7%)	
	45–60		30 (17.8%)	20 (22.7%)	
	≥ 60		6 (3.6%)	2 (2.3%)	
Classification	RR-TB by Xpert	n, %	56 (33.3%)	1 (1.1%)	<0.005
	RR-TB		49 (29.2%)	-	
	MDR-TB		63 (37.5%)	84 (95.5%)	
	XDR TB		-	3 (3.4%)	
Diagnosis	Xpert MTB/RIF	n, %	81 (48.2%)	26 (29.6%)	0.022
	MTBDRplus LPA		75 (44.6%)	54 (61.4%)	
	DST		6 (3.6%)	2 (2.3%)	
	Unknown		6 (3.6%)	6 (6.8%)	
AFB smear microscopy status	Negative	n, %	24 (14.3%)	8 (9.1%)	0.155
	Positive		40 (23.8%)	30 (34.1%)	
	Unknown		104 (61.9%)	50 (56.8%)	
Site of disease	Pulmonary	n, %	165 (98.2%)	83 (94.3%)	0.089
	Extra-pulmonary		3 (1.8%)	5 (5.7%)	
Diagnosed at	Clinic/CHC	n,%	113 (67.3%)	38 (43.2%)	0.099
	Hospital		55 (32.7%)	50 (56.8%)	
HIV status	Positive	n,%	149 (88.7%)	Not available	Not
	Negative/Unknown		19 (11.3%)		applicable
Location of residence	Distance from diagnosing/referral facility to residence, km	Median, IQR	12.0 (11.2–54.5) (n = 161)	54.2 (11.3–69.5) (n = 88)	0.726
	Distance from DR-TB treatment facility to residence, km	Median, IQR	13.0 (7.8–29.0) (n = 162)	11.1 (7.6–12.0) (n = 87)	0.091
Diagnosis site	Proportion diagnosed and treated at the same site	n, %	43 (25.6%)	0 (0%)	Not applicable

RR-TB rifampicin resistant tuberculosis; MDR-TB multi-drug resistant TB; XDR TB extensively drug resistant TB; AFB acid fast bacilli; LPA line probe assay; phenotypic DST drug susceptibility testing; HIV human immunodeficiency virus; km kilometer; CHC community health centers IQR inter-quartile range

For the sample as a whole, the median time from sputum collection to diagnosis was 26 days (IQR 7–36) and from sputum collection to treatment initiation 33 days (IQR 12–52; n = 256). Time from sputum collection to treatment initiation varied by type of treatment center: 42 days (IQR 29–55) and 22 days (9–50) for inpatient (n = 88) and outpatient sites (n = 168), respectively (p = 0.03). This interval also varied by diagnostic method for diagnosis of rifampicin resistance (e.g. Xpert MTB/RIF vs LPA and phenotypic DST): 17 days (9–47), 38 days (23–54), and 81 days (49–115) for Xpert MTB/RIF (n = 107), LPA (n = 129), and phenotypic DST (n = 8), respectively (p = 0.002). Table 3 summarizes median time from sputum collection to treatment initiation, by treatment site and diagnostic method.

**Table 3:** Median time from sputum collection to treatment initiation, by treatment site and diagnostic method and proportion initiating treatment by treatment site (n = 256).

	All Xpert MTB/RIF	GenoType MTBDRplus line probe assay	Phenotypic drug susceptibility testing	P value
**Median time from sputum collection to treatment initiation**				
**DR-TB treatment site**				
All (n = 256)	17 (9–47)	38 (23–54)	81 (49–115)	0.002
Outpatient (n = 168)	13 (9–28)	41 (6–62)	75 (28–133)	0.01
Inpatient (n = 88)	49 (33–64)	37 (29–46)	89 (81–97)	0.04
**AFB smear microscopy status**				
Positive (n = 70)	15 (1–42)	32 (7–42)	81 (75–107)	0.003
Negative (n = 32)		54 (45–62)	97 (28–133)	0.316
**Proportion initiating DR-TB treatment by DR-TB treatment site**				
Outpatient	N = 107	N = 129	N = 8	
Helen Joseph Hospital (outpatient)	18 (16.8%)	46 (35.7%)	4 (50.0%)	
Charlotte Maxeke Hospital (outpatient)	35 (32.7%)	21 (16.3%)	1 (12.5%)	
South Rand Hospital (outpatient)	28 (26.2%)	8 (6.2%)	1 (12.5%)	
Inpatient				
Sizwe Tropical Disease Hospital	26 (24.3%)	54 (41.9%)	2 (25.0%)	

Only ten percent (25/256) of patients diagnosed with RR-TB initiated treatment within five days, which is the national target [21]. Those starting treatment at the inpatient facility were more likely to initiate after five days (RR 1.15 95% CI 1.08–1.23). More patients at the outpatient facilities started treatment within five days than at the inpatient facility (1% [1/88] vs 14% [24/168]), though the proportion achieving this target, 14%, was very low even at the decentralized facilities.

### Factors predicting treatment initiation and mortality

We demonstrate that compared to those diagnosed by Xpert MTB/RIF, patients diagnosed by phenotypic DST were less likely to initiate treatment (sHR 0.39 95% CI 0.20–0.75). Patients diagnosed with XDR-TB were also less likely to link to care (sHR 0.38 95% CI 0.15–0.98). Distance from residence to referring facility was a significant predictor of treatment initiation (Table 4).

**Table 4:** Subdistribution hazard regression to identify predictors treatment initiating among patients diagnosed with RR-TB in the COJ between July 2011 and June 2012.

			Proportion initiating TB treatment (n = 256)		
Characteristic			Treatment initiation/N (%)	Crude sHR^ (95% CI)	Adjusted sHR^ (95% CI)
Gender	Female	130/287 (45.3%)		1.0	1.0
	Male	126/307 (41.0%)		0.87 (0.68–1.12)	0.88 (0.68–1.14)
Age, years	< 30	73/162 (45.1%)		1.0	1.0
	30–45	125/308 (40.6%)		0.92 (0.69–1.24)	0.99 (0.73–1.33)
	45–60	50/105 (47.6%)		1.13 (0.79–1.63)	1.23 (0.84–1.79)
	≥ 60	8/19 (42.1%)		1.09 (0.49–2.43)	1.20 (0.50–2.88)
Classification	RR-TB by Xpert	147/320 (45.9%)		0.91 (0.65–1.26)	0.82 (0.54–1.24)
	RR-TB	57/158 (36.1%)		1.04 (0.76–1.41)	1.10 (0.79–1.53)
	MDR-TB	49/102 (48.0%)		1.0	1.0
	XDR TB	3/14 (21.4%)		0.39 (0.13–1.19)	0.38 (0.15–0.98)
Diagnosis	Xpert MTB/RIF	107/258 (41.5%)		1.0	1.0
	LPA	129/281 (45.9%)		0.97 (0.74–1.27)	0.79 (0.55–1.16)
	DST	8/30 (26.7%)		0.43 (0.22–0.87)	0.39 (0.20–0.75)
	Unknown	12/25 (48.0%)		1.08 (0.59–1.96)	0.85 (0.44–1.65)
AFB smear microscopy status	Positive	70/144 (48.6%)		1.35 (0.89–2.04)	
	Negative	32/78 (41.0%)		1.0	
	Unknown	154/372 (41.4%)		1.21 (0.84–1.74)	
Site of disease	EPTB	8/13 (61.5%)		1.0	1.0
	Pulmonary	248/581 (42.7%)		0.70 (0.38–1.26)	0.82 (0.40–1.67)
Distance from residence to referring facility	Near	57/119 (47.9%)		1.60 (1.15–2.24)	1.64 (1.17–2.30)
	Intermediate	118/191 (61.8%)		1.0	1.0
	Far	74/92 (80.4%)		3.47 (2.63–4.56)	3.39 (2.54–4.52)

sHR subdistribution hazard ratio

^sHR from competing risk regression accounting for death. CI confidence interval; EPTB extra-pulmonary TB; AFB acid fast bacilli; RR-TB rifampicin resistant TB; MDR-TB multi-drug resistant TB; XDR extensively drug resistant TB; LPA line probe assay; phenotypic DST drug susceptibility testing; Reference = HR 1.00

Time to treatment initiation varied by diagnostic method, and this then may explain some of the differences observed in mortality before treatment initiation. Those diagnosed by LPA (HR 2.57 95% CI 1.31–5.03) and phenotypic DST (HR 3.98 95% CI 1.58–9.99) were more likely to die before treatment initiation, compared to those diagnosed by Xpert MTB/RIF. Patients classified as having XDR TB (HR 3.35 95% CI 1.33–8.42) were more likely to die before treatment initiation compared to those with MDR-TB. Conversely, those classified as having rifampicin mono-resistance were less likely to die before treatment initiation (HR 0.51 95% CI 0.26–1.00). Interestingly, patients who lived near to (sHR 1.64 95% CI 1.17–2.30) and those who lived far (sHR 3.39 95% CI 2.54–4.52) from the diagnosing/referring facility were more likely to initiate treatment. Those who lived greater distances from the diagnosing/referring facility were also less likely to die before treatment initiation (far vs intermediate HR 0.09 95% CI 0.02–0.36) (Table 5).

**Table 5:** Unadjusted and adjusted predictors of mortality among patients diagnosed with RR-TB in the COJ between July 2011 and June 2012.

		Mortality (n = 104)		
Characteristic		Mortality/N (%)	Crude Hazard Ratio (95% CI)	Adjusted Hazard Ratio (95% CI)
Gender	Female	46/287 (16.0%)	1.0	1.0
	Male	58/307 (18.9%)	1.14 (0.77–1.69)	0.98 (0.65–1.48)
Age, years	< 30	20/162 (12.4%)	1.0	1.0
	30–45	63/308 (20.5%)	1.85 (1.09–3.13)	1.94 (1.13–3.33)
	45–60	18/105 (17.1%)	1.55 (0.81–2.98)	1.52 (0.77–3.01)
	≥ 60	3/19 (15.8%)	1.40 (0.41–4.75)	1.26 (0.37–4.35)
Classification	RR-TB by Xpert	23/158 (14.6%)	0.93 (0.57–1.51)	1.80 (0.87–3.76)
	RR-TB	13/102 (12.8%)	0.61 (0.32–1.16)	0.51 (0.26–1.00)
	MDR-TB	61/320 (19.1%)	1.0	1.0
	XDR TB	7/14 (50.0%)	2.86 (1.23–6.62)	3.35 (1.33–8.42)
Diagnosis	Xpert MTB/RIF	34/258 (13.2%)	1.0	1.0
	LPA	56/281 (19.3%)	1.41 (0.92–2.17)	2.57 (1.31–5.03)
	DST	11/30 (36.7%)	2.75 (1.32–5.73)	3.98 (1.58–9.99)
	Unknown	3/25 (12.0%)	0.85 (0.26–2.76)	1.43 (0.39–5.16)
AFB smear microscopy status	Positive	26/144 (18.1%)	0.78 (0.43–1.43)	
	Negative	19/78 (24.4%)	1.0	
	Unknown	59/372 (15.9%)	0.65 (0.38–1.11)	
Site of disease	EPTB	1/13 (7.7%)	1.0	1.0
	Pulmonary	103/581 (17.7%)	2.55 (0.36–18.30)	3.11 (0.43–22.61)
Distance from residence to referring facility	Near	25/119 (21.0%)	0.98 (0.62–1.57)	0.99 (0.62–1.62)
	Intermediate	21/191 (11.0%)	1.0	1.0
	Far	2/92 (2.2%)	0.09 (0.02–0.36)	0.09 (0.02–0.36)

CI confidence interval; EPTB extra-pulmonary TB; AFB acid fast bacilli; RR-TB rifampicin resistant TB; MDR-TB multi-drug resistant TB; XDR extensively drug resistant TB; LPA line probe assay; phenotypic DST drug susceptibility testing; Reference = HR 1.00

## Discussion

In this study, fewer than half (43.0%) the patients diagnosed with DR-TB in the City of Johannesburg in 2011–2012 initiated appropriate treatment within six months of diagnosis despite the quadrupling of facilities offering DR-TB treatment under the new decentralization framework–from one inpatient facility to one inpatient plus three outpatient facilities. The proportion of patients who initiated treatment within six months (43.0%) is similar to national estimates reported for South Africa in the 2013 WHO Global TB report (6 494 cases started on MDR-TB treatment/15 419 cases of laboratory confirmed MDR-TB for 2012; 42%) [22]. Pre-treatment loss to follow-up among drug sensitive patients in Africa ranges from 6 to 38%, and is similar among patients with drug resistant TB [23–26]. We showed high pre-treatment loss of 17.8% and many patients (17.5%) died before DR-TB treatment could be initiated. Only 3 out of 5 patients diagnosed with DR-TB (60.9%) could be traced and referred for treatment. Although decentralization and the implementation of Xpert MTB/RIF for the diagnosis of TB and rifampicin resistance has resulted in a significant reduction in time to treatment initiation as reported in previous studies [26], these programmatic changes did not solve the problem of loss of patients between diagnosis and treatment initiation seen in our cohort. This initial loss from care has contributed to lack of improvement in treatment outcomes [27]. This is an area where additional health system strengthening is required.

For the study period, we report a median time to treatment initiation of 33 days, which is consistent with what has been reported elsewhere in the country following implementation of decentralized treatment. Results from Khayelitsha, Cape Town report that the introduction of LPA during 2007–2008 was associated with a decrease in time to treatment initiation from 76 to 50 days. Decentralization during 2008–2011 saw a further reduction to 28 days (IQR 16–40) [26]. Our results show that time to treatment initiation varied by diagnostic method and whether the patient was smear positive or negative, and the reason for the test. Time from sputum collection to diagnosis differed by diagnostic method, which in turn delayed reporting, referral, and treatment initiation. Xpert MTB/RIF was implemented as the first-line diagnostic test for tuberculosis in South Africa during the study period, as such some patients some patients in the early study period would have been diagnosed under the culture and LPA diagnostic algorithm. Time to treatment initiation has improved significantly following implementation of Xpert MTB/RIF in South Africa, from a median delay of 28–62 days [26,28] with the use of Genotype MTBDRplus line probe assay, to 8–10 days since implementation of Xpert MTB/RIF [26,29].

Negative sputum smears are also correlated with delays for TB treatment in those diagnosed by LPA [30,31] because LPAs are generally performed on culture, rather than directly on specimen, in smear negative cases, resulting in significant delays in therapy (24 days vs 62 days) [32]. In addition to the diagnostic method used, the burden of TB in the setting may also contribute to delays in initiation. High numbers of RR-TB positive cases may result in delays in home visits or reporting of laboratory results, which can result in delayed or inappropriate treatment and missed opportunities to prevent transmission. In our setting, Xpert MTB/RIF has been used as the initial diagnostic test since late 2011 for TB. Smear and culture are ordered if Xpert MTB/RIF negative and the patient is still symptomatic for TB. LPA is done on smear positive sputa or on MTB+ culture isolates whereas if smear negative, a culture is grown and then LPA is done on the culture, which in turn delays reporting, referral, and treatment initiation. Our study coincides with the rollout of Xpert MTB/RIF with laboratories in high burden sub-districts receiving Xpert MTB/RIF machines first. Xpert MTB/RIF implementation was accompanied by considerable operational and logistical challenges [9], and as a result not all sites had access to Xpert MTB/RIF during the early part of the study. Full, nation-wide, capacitation was reached in September 2013, however in the City of Johannesburg full capacity was reached in early 2012 [15].

In our study, patients diagnosed by LPA and phenotypic DST were more likely to die before treatment initiation. The median time from sputum collection to treatment initiation for patients diagnosed by these methods—38 days and 81 days for LPA and phenotypic DST, respectively, compared to just 17 days for diagnosis by Xpert—may in itself place patients at risk: in a 2010 study from KwaZulu Natal, South Africa, 40% of MDR-TB and 51% of XDR TB patients died within 30 days of sputum collection [7]. A recent study demonstrated the impact of reducing time to MDR-TB treatment initiation on treatment outcomes. The study showed that time to MDR-TB treatment initiation was lower in the group diagnosed using the LPA-based algorithm compared to those diagnosed with the culture-based algorithm (50 and 66 days) which resulted in better treatment outcomes, both in terms of treatment success (65.2% vs 44.8%) and mortality (7.6% vs 15.9%) [32]. Prior to implementation of Xpert MTB/RIF as the first line diagnostic test, phenotypic DST and LPA were done on patients suspected of having DR-TB due to prior treatment failure, default, or contact with a person with known DR-TB [14]. As a result the groups of patients diagnosed in the previous algorithm may reflect a sicker population of patients with longer standing undiagnosed/untreated tuberculosis at higher risk of early mortality. As a result the group diagnosed by Xpert MTB/RIF may not be equivalent than those diagnosed by LPA.

We found that patients who lived near (<11.2 km) to the diagnosing/referring facility were more likely to initiate treatment compared to those at a middle or intermediate distance. This was supported by the finding that patients who lived in the same COJ region as the diagnosing/referring facility (RR 1.24 95% CI 0.92–1.67) or the same region as one of the treatment facilities (RR 1.47 95% CI 1.14–1.90) were more likely to initiate treatment. Contradictory to other reports, we also found that patients who lived far (≥54.3 km) from the diagnosing/referring facility were more likely to initiate treatment. Therefore, according to distance from diagnosing/referring facility, we found the highest rates of initiation among two groups, those who live very close and those who live very far, with less initiation for those at a middle distance from the diagnosing/referring facility (e.g. not close to work or home). There are a number of possible factors that may help explain why patients who live very far from the diagnosing/referring facility are more likely to initiate treatment. There could be a component of selection bias as those patients located far from the urban center who manage to seek out care and get diagnosed with DR-TB may reflect a sub-set of patients more likely to engage in care. Also, patients who are still well enough to work may be using the facility closest to their employment, as facilities are only open during working hours, rather than near their residential address (which is typically located outside of the cities and places of work). Another contributing factor may be the use of public transportation (e.g. patients may choose to travel to a more distant clinic as it requires less taxi switches, cost and time). Some other patient related factors that may contribute to delaying treatment include first consulting with a traditional healer, financial constraints, stigmatization and misperceptions about TB causes and symptoms [30, 33–36]. Type of health care provider (e.g. initial visit to a specialized TB facility) may be an important factor for patients seeking care [37,38]. Provider factors such as infrastructure of health services, diagnostic facilities, availability of trained staff, quality of services and effective supervision may persuade patients to travel long distances to access and utilize healthcare services [39] Further exploration of this unexpected finding is required. The current study was retrospective and was not designed to explore the patients’ health seeking behaviour.

COJ tracing successfully referred 60.9% and traced an additional 21.5% (n = 128; deaths and transfer out) of patients diagnosed with DR-TB. Consistent with other reports from this region, 79% of those referred initiated treatment [40]. Guidelines recommend that tracing should be completed in 3–5 days; we observed a median of 14 days. Reducing this delay, by investing more in the efforts of TB district coordinators, may increase the proportion of patients referred and reduce pre-treatment mortality. It is also important to understand why close to a fifth (17.8%) of patients who were referred did not initiate treatment within COJ in the six-month follow up interval of study observation. Some may have started treatment at another facility outside of COJ or after six months, but existing tracking systems do not allow this to be determined. An additional 7% (n = 29; 8 diagnosed by Xpert MTB/RIF and 21 by LPA) of patients who were referred had a treatment initiation date prior to the diagnosis date. Since these are likely to represent a previous episode (i.e. initiation date median 3.2 months IQR 1.3–6.8 before the diagnosis date), they were assumed not to have initiated treatment for this RR-TB episode.

Results should be considered in light of the study limitations. First, as this was a retrospective medical register review there were some variables, such as HIV status, travel time or travel cost and previous history of TB, which could not be included in the analysis. Moreover, although death dates were available for most of those who died (93%), dates of other outcomes (e.g. moved or transferred) were reported for only a third of those concerned (34%), requiring us to use the date of last contact with patient as recorded by the district coordinator as a proxy date. Since death is ascertained from family reports and passive tracing by the district TB coordinator, it is possible that some of those lost (n = 104) may have died, resulting in an underestimation of mortality prior to treatment initiation. Although disease classification was recorded from the COJ register, there may have been some misclassification due to inadequate second-line resistance testing, and as such, several people classified as RR-TB may be MDR-TB or (pre) XDR TB. Though we attempted to verify the diagnostic test and data, using the COJ register and NHLS laboratory reports, diagnosis method and the date of diagnosis may have been incorrect in some instances. For example, if the initial diagnostic test (e.g. Xpert MTB/RIF) was missing we used LPA and the date of the LPA as the diagnostic test.

Second, since the starting point of the study was the COJ register (NHLS sends all RR-TB results back to the diagnosing clinic and to the COJ TB coordinator who records the result and assigns a TB case registration number) we could not ascertain if all laboratory diagnosed RR-TB cases were registered in the COJ register and therefore included in the study. Albeit minimal, this may have led to an underestimation of the number of cases of laboratory confirmed RR-TB, in the COJ, for the study period. In addition, we relied on the COJ register for linkage outcomes and dates of linkage outcomes for SDTH patients in the cohort and did not access the DR-TB register or EDRweb for this treatment site. Finally, although we attempted to match eligible patients using the DR-TB case registration number, in some cases this was missing in the electronic registers at the site, leading us to underestimate the proportion of patients who initiated treatment. Although we used various combinations of patient first name, surname, date of birth and gender to match patients in the COJ register to electronic registers at the sites, we cannot be certain that no patients were missed.

## Conclusion

Despite these limitations, our data indicate very high rates of failure to initiate appropriate RR-TB treatment for patients diagnosed with drug-resistant TB in the City of Johannesburg. Though it is surely a step in the right direction, offering treatment at decentralized sites alone is not sufficient; improvements in linking patients diagnosed with RR-TB to effective treatment remains a high priority.
